# Effects of Dietary Interventions on Cognitive Outcomes

**DOI:** 10.3390/nu17121964

**Published:** 2025-06-09

**Authors:** Judith Charbit, Jean-Sébastien Vidal, Olivier Hanon

**Affiliations:** Service Gérontologie Hôpital Broca, Université Paris Cité, AP-HP, F-75013 Paris, France; judith.charbit@aphp.fr (J.C.); jean-sebastien.vidal@aphp.fr (J.-S.V.)

**Keywords:** vitamins, nutrients, dietary interventions, cognitive assessment, cognitive function, mental health, cognitive aging, mild cognitive impairment, metabolic disorders, review

## Abstract

Cognitive aging is a complex, multifactorial process influenced by genetic, metabolic, and environmental factors. Among modifiable risk factors, nutrition has emerged as a promising target to preserve cognitive function. This review provides a comprehensive overview of the impact of dietary interventions—including specific nutrients and dietary patterns—on cognitive domains (memory, executive function, global cognition) and mental health. Recent findings: multinutrient supplementation, particularly combinations of B vitamins and omega-3 fatty acids, appears beneficial for episodic memory, especially in individuals with metabolic risk or early cognitive impairment. Antioxidant-rich diets and the MIND diet are consistently associated with better memory and global cognitive outcomes in observational studies. Effects on executive function and mental health remain heterogeneous, although subgroups such as those with mild cognitive impairment or depression may derive benefit. Evidence from interventional studies remains limited by methodological variability. In conclusion, dietary interventions offer a safe and promising approach to support cognitive health in aging. Future research should focus on developing personalized, multidomain prevention models tailored to individual risk profiles.

## 1. Introduction

### 1.1. Normal Cognitive Aging and Cognitive Health

Cognitive aging is a natural process marked by a gradual decline in abilities such as memory, processing speed, and conceptual reasoning. Recent neurological research has identified reductions in gray and white matter, as well as neurotransmitter levels, as contributing factors to these changes [1]. Importantly, normal cognitive decline does not interfere with daily functioning. Hendrie et al. define cognitive health as “the development and preservation of the multidimensional cognitive structure that allows older people to maintain social connectedness, an ongoing sense of purpose and the abilities to function independently, to permit functional recovery from illness or injury, and to cope with residual functional deficits” [2]. When cognitive impairments begin to interfere with daily activities, it becomes essential to investigate potential underlying causes, including neurodegenerative diseases such as dementia. The rate of cognitive decline varies significantly between individuals, influenced by genetic, medical, and environmental factors that may accelerate the process.

### 1.2. The Role of Nutrition in Cognitive Health

Nutrition plays a vital role in brain development, beginning in pregnancy and continuing throughout life. Key nutrients such as proteins, fats, iron, zinc, iodine and folate are essential for supporting brain development (for example neurogenesis, the process of generating new neurons, may be influenced by nutritional factors), maintaining cognitive function, and mitigate neuropsychiatric disorders [3]. Studies have established a connection between diet quality and cognitive performance, with growing evidence supporting the role of nutrition in preventing cognitive decline in older adults. For instance, research on Australian older adults demonstrated that better diet quality was significantly linked to enhanced memory and attention [4]. Conversely, a cross-sectional analysis of NHANES 2011–2014 showed that the consumption of ultra-processed foods, characterized by high levels of saturated fatty acids and simple sugars, was associated with diminished cognitive performance in older adults without pre-existing conditions [5]. This dietary pattern is now recognized as an environmental risk factor for Alzheimer’s disease, as it promotes insulin resistance, neuronal inflammation, accumulation of insoluble proteins in the brain, and microbiota imbalances. In particular, chronic consumption of ultra-processed foods rich in saturated fats and refined sugars has been linked to increased cerebral deposition of insoluble amyloid-beta (Aβ) and hyperphosphorylated tau proteins—two neuropathological hallmarks of Alzheimer’s disease. These abnormal protein aggregates disrupt synaptic function and neuronal communication, contributing to cognitive decline. Conversely, diets rich in antioxidants, polyphenols, and omega-3 fatty acids have been associated with reduced amyloid burden and tau pathology, possibly by enhancing proteostasis mechanisms, reducing oxidative stress, and modulating neuroinflammation [6].

### 1.3. Preventing Dementia

Dementia is a leading cause of disability-adjusted life years lost among older adults. With 152 million cases projected worldwide by 2050, it presents a growing medical, social, and economic challenge [7]. Currently, there are no treatments capable of significantly slowing or reversing cognitive decline, aside from the modest effects observed with lecanemab in early-stage Alzheimer’s disease (AD) [8]. Modifiable risk factors, such as depression, hypertension, type 2 diabetes, obesity, smoking, physical inactivity, and low educational levels, play a pivotal role in preventing dementia. The 2024 Lancet Commission on dementia prevention emphasizes addressing these risks through interventions to enhance overall health [9].

### 1.4. Most Studied Dietary Interventions

*B vitamins*: These are crucial for homocysteine metabolism and have been studied for their potential role in cognitive function. Hyperhomocysteinemia is a well-established independent risk factor for cognitive impairment and dementia. Folate (abundant in leafy green vegetables), vitamin B6 (found in grains, legumes, and nuts), and B12 (primarily sourced from dairy, meat and other animal products) have been examined for their ability to lower homocysteine levels, reduce oxidative stress, and support DNA methylation [10]. Numerous interventional studies have investigated the influence of B vitamin supplement intake on cognitive function.

*Omega-3 long-chain polyunsaturated fatty acids (PUFAs)*: These are essential fatty acids, including eicosapentaenoic acid (EPA), docosahexaenoic acid (DHA) and alpha-linolenic acid (ALA), essential for brain health and commonly found in fish, nuts, and green leafy vegetables. They seem to support cognitive function and neuroprotection.

*Antioxidants:* Vitamins C and E, carotenoids, and polyphenols, along with minerals like selenium and zinc, act as antioxidants that protect the brain from free radical damage. This oxidative stress, a key risk factor for cognitive decline, is particularly relevant to AD. Many studies have explored the association between cognitive function and antioxidant supplementation.

*Mediterranean-dash diet intervention for neurological delay (MIND):* The MIND diet combines elements of the Mediterranean and DASH diets (Dietary Approaches to Stop Hypertension), both known for their cardiovascular benefits. While the Mediterranean diet emphasizes plant-based foods, fish, olive oil, and low red meat intake to reduce inflammation and brain aging, the DASH diet focuses on fruits, vegetables, whole grains, and low sodium to support vascular health. Together, the MIND diet enhances neuroprotection through its emphasis on antioxidants, B vitamins, and omega-3 fatty acids.

*Ketogenic diet (KD)*: This is characterized by high fat and low carbohydrate intake and increases ketone body production. It has been shown to reduce neuroinflammation and amyloid toxicity in animal studies and may support cognition. Different types of KDs exist: the classical KD, the modified Atkins diet (MAD) and the MCT diet, in which fats are provided with medium-chain triglyceride (MCT) intake.

*Caloric restriction (CR) and intermittent fasting (IF)*: Both strategies involve reducing caloric intake while maintaining essential nutrient adequacy. They have demonstrated neuroprotective effects in animal models and show promising, although mixed, cognitive benefits in human studies—particularly in relation to memory and executive function. Importantly, intermittent fasting encompasses various regimens, including time-restricted feeding (e.g., eating within 8–10-h windows), alternate-day fasting, and prolonged nightly fasting, each with distinct metabolic effects. Emerging evidence suggests that prolonged nightly fasting may confer benefits on global cognition and metabolic regulation. Moreover, IF has been associated with improvements in cardiovascular and metabolic health—including reduced insulin resistance, blood pressure, and inflammation—which may indirectly support brain health and cognitive resilience.

The functioning of the human brain relies on a dynamic interaction between inherited genetic factors and external environmental influences, including diet. Nutrition plays a critical role in sustaining brain performance while also contributing to the prevention and treatment of mental disorders [11]. This narrative review provides a comprehensive overview of the most recent literature on the effects of major nutritional interventions on cognitive function and mental health. It does not aim to be exhaustive, but rather to synthesize key findings and emerging trends in this evolving field.

## 2. Methodology

This narrative review was conducted to provide a broad and up-to-date synthesis of the most recent literature on the effects of dietary interventions on cognitive function and mental health in older adults. The objective was not to perform a systematic review or meta-analysis, but rather to offer a structured overview of major nutritional strategies studied in relation to cognition, based on the most relevant and methodologically robust studies.

The literature search was carried out in the PubMed and Scopus databases, using combinations of the following keywords: “diet”, “nutrition”, “cognition”, “cognitive decline”, “executive function”, “memory”, “dementia”, “MCI”, “mild cognitive impairment”, “depression”, “mental health”, “omega-3”, “B vitamins”, “polyphenols”, “antioxidants”, “MIND diet”, “Mediterranean diet”, “ketogenic diet”, “intermittent fasting” and “caloric restriction.” Filters were applied to select English-language peer-reviewed articles published between January 2018 and March 2025.

We included observational studies, randomized controlled trials (RCTs), and meta-analyses that reported cognitive or mental health outcomes in adults, with a focus on interventions targeting nutrients, dietary patterns, or caloric restriction strategies. Studies that investigated children or patients with acute neurological conditions were excluded.

To ensure clarity and thematic consistency, cognitive outcomes were classified into four main domains: memory, executive function, global cognition, and mental health. For each domain, evidence was summarized by type of intervention (nutrients, dietary patterns, or caloric restriction), and stratified by study design (observational, interventional, or meta-analytical). This structure was chosen to facilitate comparison between interventions and highlight patterns of evidence strength and consistency.

## 3. Results

### 3.1. Memory

The hippocampus plays a critical role in learning, memory consolidation, and emotional regulation. Its unique ability to support neurogenesis throughout life makes it particularly sensitive to nutritional and metabolic influences. Age-related decline in hippocampal function is associated with impairments in episodic and spatial memory, with episodic memory typically declining earlier than semantic memory. Nutritional interventions that promote synaptic integrity, neurotrophic factor expression, and reduced oxidative stress may help preserve memory performance in aging populations [1,11] (Table 1).

#### 3.1.1. B Vitamins and Memory

Observational studies have highlighted potential associations between B vitamin intake and memory performance. For example, a cross-sectional analysis of the NHANES 2011–2014 cohort revealed that higher vitamin B2 intake was significantly associated with improved performance on the CERAD memory test in older adults [12]. Similarly, the REACH cohort in New Zealand reported a modest but significant link between elevated glycine concentrations (influenced by B vitamin metabolism) and better episodic memory [13].

Randomized controlled trials (RCTs) provide more nuanced findings. The COSMOS-Web trial showed that daily multivitamin supplementation (including B vitamins) led to improved immediate recall as measured by the ModRey test after one year, with effects persisting over three years [14]. However, not all multicomponent supplements have proven effective results. A study by Young et al. evaluated the impact of a combination supplement containing B vitamins, *Bacopa monnieri*, and *Ginkgo biloba* in healthy adults, and found no significant improvement in memory performance [16].

The 2023 meta-analysis by Fairbairn et al. demonstrated that combined supplementation with B vitamins and omega-3 PUFAs significantly enhanced episodic memory in older adults [15]. However, supplementation with vitamin B12 alone appears less effective: a meta-analysis by Markun et al. found no significant memory benefits from B12, whether administered alone or in combination with folate and B6 [17].

In conclusion, while individual B vitamin supplementation—particularly B12—may have limited efficacy, multinutrient strategies that include B vitamins and omega-3 fatty acids appear more promising in supporting episodic memory performance in older adults.

#### 3.1.2. Omega-3 LC-PUFAs and Memory

The role of long-chain omega-3 polyunsaturated fatty acids (LC-PUFAs), including DHA and EPA, has been extensively explored.

Observational findings from NHANES show positive associations between higher dietary omega-3 intake and verbal episodic memory in older adults [18].

Interventional studies have yielded mixed results. For instance, Maltais et al. [20] reported that EPA and DHA taken for six months improved episodic memory, particularly in individuals with lower baseline performance. Conversely, larger trials [22,23] found no significant effects on verbal memory, suggesting variability depending on population characteristics, dosage, and duration.

In specific populations with metabolic comorbidities, such as older adults with stable coronary artery disease, high-dose supplementation with EPA and DHA over 30 months led to significant improvements in memory performance, as demonstrated by the Rey Auditory Verbal Learning Test (RAVLT) in the study by Malik et al. [21].

Meta-analytic data further support these observations: a 2020 review by Alex et al. reported a significant positive effect of omega-3 LC-PUFAs on memory function [19], while Fairbairn et al. demonstrated that multinutrient interventions combining omega-3 PUFAs with B vitamins significantly improved episodic memory compared to placebo [15].

In summary, although findings are somewhat heterogeneous, the body of evidence supports a beneficial role of omega-3 fatty acids in maintaining or enhancing memory performance, particularly episodic memory, in older adults. These effects appear most pronounced in multinutrient formulations or among individuals with lower cognitive baselines or metabolic comorbidities.

#### 3.1.3. Antioxidants and Memory

Oxidative stress is a well-established contributor to neuronal damage and memory impairment.

Observational studies consistently report a positive association between higher antioxidant intake and memory outcomes. In the Rush Memory and Aging Project, elevated dietary flavonol consumption was linked to a significantly slower annual decline in episodic memory over approximately seven years [24]. Similarly, a French cohort study showed that greater midlife polyphenol intake was associated with superior verbal memory performance 13 years later [25].

Interventional studies largely support the memory-enhancing potential of antioxidants, although results vary across compounds and populations. Trials with polyphenol-rich extracts—such as wild blueberry juice, grape–blueberry formulations, or 6-MSITC (from wasabi)—consistently reported improvements in episodic memory, particularly in older adults with low baseline performance or subjective memory complaints [26,28,29]. Some studies, like that by Lopresti et al. [27], demonstrated benefits on episodic memory using multi-compound antioxidant supplementation, although effects on other memory domains were inconsistent. Conversely, other trials, including those using cocoa extract or elderberry juice, did not observe significant cognitive gains [34,35]. In populations with metabolic comorbidities or mild cognitive impairment (MCI), findings are sparse but suggest potential benefits when antioxidant interventions also improve metabolic function, as seen with resveratrol in older women [30,35].

Meta-analytic evidence aligns with these findings. A COSMOS-based analysis reported significant improvements in episodic memory among older adults receiving daily multivitamin–mineral supplementation [31]. Curcumin also demonstrated consistent benefits for memory performance, in healthy older adults [32]. While some reviews, such as that by Khorshidi [36], found no significant cognitive effects of resveratrol alone, broader meta-analyses suggest that polyphenols as a group are positively associated with improvements in episodic memory [33].

In summary, antioxidant interventions—particularly polyphenol-rich and multi-ingredient formulas—appear to benefit episodic memory in older adults, especially those with subtle impairments. Evidence remains limited in MCI and metabolically at-risk populations, warranting further research.

#### 3.1.4. MIND Diet and Memory

Observational studies suggest that adherence to the MIND diet may positively influence memory performance in older adults. A Dutch cross-sectional study in elderly individuals without dementia found that greater adherence to the MIND diet correlated with higher memory scores [37]. Similarly, in a cognitively healthy Brazilian cohort, closer adherence was associated with better memory recall, while no such benefit was observed in individuals with MCI or AD [38]. In the Nurses’ Health Study, long-term adherence to the MIND diet was modestly linked with better verbal memory in women [39].

Interventional studies remain limited and show mixed results. A 3-month RCT involving 40 overweight women reported improved verbal learning and recall in participants following the MIND diet compared to controls [40]. However, other short-term feasibility studies have not shown significant improvements in objective memory measures, despite enhanced dietary quality [41].

No meta-analysis to date has focused exclusively on the effects of the MIND diet on memory outcomes.

In conclusion, current evidence from observational studies supports a potential role for the MIND diet in preserving memory in cognitively healthy older adults. Nonetheless, data from randomized trials remain scarce, and further high-quality research is needed to confirm its efficacy.

#### 3.1.5. Ketogenic and Low-Carbohydrate Diets and Memory

Observational studies provide initial support for a relationship between low-carbohydrate dietary patterns and memory performance. For instance, Wang et al. reported that higher adherence to a low-carbohydrate diet was positively associated with better memory scores in older adults [42].

RCTs suggests that ketogenic and low-carbohydrate diets may enhance memory function, particularly in individuals with MCI. Studies by Krikorian et al. [43] and Fortier et al. [44] demonstrated that increased ketone availability was associated with improved memory recall. Supplementation with MCTs, which promote ketosis, also yielded gains in episodic memory. However, in a feasibility trial among AD patients, Buchholz et al. found no significant cognitive benefits, likely due to challenges with dietary adherence [45].

In conclusion, ketogenic and low-carbohydrate diets show promise for memory enhancement, particularly in MCI populations. Nevertheless, limitations such as small sample sizes, dietary adherence variability, and lack of long-term follow-up necessitate further research to validate these preliminary findings and assess their broader clinical applicability.

#### 3.1.6. **Dietary Restrictions (CR and IF) and Memory**

Observational studies suggest that dietary restriction may support memory preservation in aging populations. A prospective study by Ooi et al. followed older adults with MCI over 36 months and found that those who regularly or irregularly practiced IF had significantly better memory performance—as measured by the RAVLT T-score—than those who did not fast [46].

Evidence from RCTs provides variable but supportive findings. Keawtep et al. reported no significant cognitive benefits from IF alone in obese postmenopausal women. However, physical–cognitive exercise, with or without dietary intervention, led to meaningful improvements in memory performance [47].

Finally, a recent meta-analysis concluded that dietary restriction has a positive effect on cognitive function in individuals with MCI [48].

Overall, these findings suggest that CR and IF can benefit memory, especially in older adults at risk of cognitive decline, although responses may differ by population and intervention adherence. These strategies may be particularly relevant in metabolically compromised populations, where dietary interventions such as caloric restriction or intermittent fasting could yield dual benefits on both metabolic health and cognitive outcomes.

### 3.2. Executive Function

Executive functions encompass a set of high-level cognitive processes, including planning, cognitive flexibility, problem-solving, and inhibitory control, that are essential for goal-directed behavior. These functions, along with attentional capacities—such as sustained, selective, and divided attention—tend to decline with age, particularly after the age of 70. Understanding the impact of nutritional interventions on these cognitive domains is critical, given their importance for autonomy and quality of life in older adults [1] (Table 2).

#### 3.2.1. B Vitamins and Executive Function

Observational data indicate a positive association between higher dietary intake of certain B vitamins and executive function in older adults. For example, the NHANES 2011–2014 cohort showed that higher intake of vitamins B1 and B2 correlated with better performance on the Animal Fluency Test (AFT) [12,49].

Interventional studies offer mixed results. Zhou et al. [50] found that B12 supplementation significantly improved calculation performance and Kwok et al. [51] observed short-term benefits in executive function at 12 months, but these gains were not sustained at 24 months.

A recent meta-analysis found no effect of B12 alone and B complex supplementation on executive function [17].

In conclusion, while there is preliminary support for B12 in improving executive function, the evidence is inconsistent over longer follow-up. Higher intake of B1 and B2 is linked to better executive functioning, though causality remains to be established and underscoring the need for larger, long-term randomized trials.

#### 3.2.2. Omega-3 LC PUFAs and Executive Function

Cross-sectional analyses suggest a positive relationship between dietary intake of omega-3 PUFAs and executive function in older adults. In the 2024 NHANES study, involving 2430 participants aged 60 years and above, higher omega-3 intake was significantly associated with better executive performance [18].

Findings from RCTs remain heterogeneous. A study by Stavrinou et al. [52] in older adults with MCI demonstrated significant improvements in executive function, as measured by the Stroop Color Test, following six months of high-dose omega-3 and omega-6 PUFA supplementation combined with antioxidant vitamins. Conversely, other trials, such as that by Howe et al. [54], involving cognitively healthy older adults, did not demonstrate any significant effects on executive functioning despite prolonged DHA and EPA supplementation. Similarly, the MAPT study [55] reported null results, potentially attributable to lower omega-3 dosages or greater heterogeneity among participants. These divergent outcomes likely reflect variations in baseline cognitive profiles, supplement dosages, intervention durations, or the sensitivity of cognitive assessments employed.

A 2022 meta-analysis by Kosti et al. [53], which reviewed nine RCTs, found a dose–response relationship between omega-3 index levels and improvements in executive function. However, the variability in trial duration (2 to 36 months), participant profiles (ranging from younger adults to those with MCI or AD), and supplementation doses may have contributed to the inconsistency of results across studies.

In Conclusion, omega-3 Supplementation Appears to Confer Modest Benefits on Executive Function, Particularly at Higher Dosages and in Populations with Cognitive Vulnerability. Nonetheless, Variability in Study Design and Outcomes Highlights the Need for Further Targeted Trials Using Standardized Cognitive Endpoints and Stratified Populations.

#### 3.2.3. Antioxidants and Executive Function

Observational data suggest a positive relationship between antioxidant-rich diets and executive functioning. In a sample of older adults, higher dietary antioxidant intake and an optimal “oxidative balance score” (reflecting combined dietary and lifestyle factors) were both associated with superior performance on the Digit Symbol Substitution Test (DSST), a measure of processing speed and executive function. These associations remained significant even after adjusting for demographic and health-related variables [56]. Furthermore, among individuals aged ≥ 60, higher antioxidant intake was more strongly linked to better executive function in those with a history of stroke [57].

Interventional studies have yielded more variable results. In patients with MCI, a 12-week supplementation with elderberry juice showed a trend toward improved cognitive flexibility compared to placebo, although results were preliminary [35]. In contrast, the COSMOS trial reported no significant effects of daily multivitamin and antioxidant supplementation on executive function or attention scores over a two-year period [31]. Likewise, a 12-week trial of 6-MSITC (a bioactive compound derived from wasabi) in healthy older adults failed to produce improvements in inhibition or reasoning. Similarly, a French study found no executive function benefits from polyphenol supplementation in midlife [25].

Findings from meta-analyses corroborate these inconsistencies. A pooled analysis of curcumin trials did not demonstrate significant effects on executive function, likely due to the limited number of studies focused on this domain and small sample sizes [32]. Similarly, broader analyses of polyphenol-rich diets revealed no conclusive evidence supporting improvements in executive functioning [58,59,60].

While observational evidence suggests that antioxidant intake may support executive function—particularly in older or metabolically vulnerable populations—findings from intervention studies and meta-analyses remain inconclusive. Future trials with targeted executive outcomes and improved methodological rigor are warranted to clarify the potential cognitive benefits of antioxidants.

#### 3.2.4. MIND Diet and Executive Function

Evidence for the effects of the MIND diet on executive function is more limited and inconsistent compared to its association with memory.

In the Framingham Heart Study Offspring cohort, individuals with higher adherence to the MIND diet demonstrated better performance on measures of attention and processing speed, including faster completion times on the Trail-Making Test Part A [61]. However, findings from the Spanish PREDIMED-Plus cohort over two years showed no significant association between baseline MIND diet adherence and changes in executive function among older adults with overweight or metabolic syndrome [62].

Interventional studies have similarly yielded null results. In a 3-month controlled trial conducted in Iran, adherence to the MIND diet in middle-aged obese women did not significantly improve performance on executive tasks such as Trail-Making Test B or the Stroop interference test [40]. 

No meta-analyses to date have evaluated the impact of the MIND diet specifically on executive function.

In summary, current evidence does not support a consistent benefit of the MIND diet on executive function, with observational and interventional studies showing mixed or null findings, particularly in metabolically vulnerable populations.

#### 3.2.5. Ketogenic and Low-Carbohydrate Diets and Executive Function

Evidence regarding the impact of ketogenic and low-carbohydrate diets on executive function is limited and somewhat divergent. An observational analysis from the NHANES 2011–2014 dataset, including 2537 adults aged ≥60 years, found no significant association between low-carbohydrate diet adherence and executive function performance [42].

In a contrast, in a RCT of individuals with mild cognitive impairment, daily supplementation with ketogenic MCT over a six-month period led to significant improvements in executive function performance [44].

In summary, while observational data do not support a relationship between low-carbohydrate dietary patterns and executive function, targeted ketogenic interventions may provide executive benefits, particularly in cognitively vulnerable populations such as those with MCI.

#### 3.2.6. Dietary Restrictions (CR and IF) and Executive Function

Evidence regarding the effects of dietary restriction on executive function in individuals with obesity remains mixed. In a RCT of 92 postmenopausal women with obesity, only participants in the combined intervention group (IF and physical–cognitive exercise) demonstrated significant improvements in executive function after three months [47]. However, in a larger trial involving sedentary older adults with obesity, adding moderate to high CR to a 20-week aerobic exercise program did not result in superior executive function outcomes compared to exercise alone, either immediately post-intervention or at 18- to 24-month follow-up [64].

A smaller pilot study by Alharbi et al. investigated the combined effects of CR (CR) and nitrate-rich beetroot juice (BRJ) in overweight and obese older adults. After 14 days, participants in the CR + BRJ group showed significantly greater improvements in executive function, as measured by the Trail Making Test Part B, compared to CR alone [63].

In conclusion, dietary restriction strategies—particularly when combined with other interventions such as exercise or nitrate-rich supplementation—may offer modest executive function benefits in individuals with obesity, although findings remain inconsistent across trials.

### 3.3. Global Cognition

Global cognition refers to an overall measure of cognitive function, commonly assessed through composite scores or standardized tools such as the Mini-Mental State Examination (MMSE), Montreal Cognitive Assessment (MoCA), or Alzheimer’s Disease Assessment Scale–Cognitive Subscale (ADAS-Cog). It integrates multiple cognitive domains—memory, attention, executive function, language, and visuospatial abilities—and provides a general index of cognitive health, making it a key outcome in studies assessing cognitive aging and dementia prevention. Given its multidimensional nature, global cognition is particularly relevant in dietary intervention studies, which often aim to capture broad cognitive benefits across domains rather than isolated functions (Table 3).

#### 3.3.1. B Vitamins and Global Cognition

Evidence regarding the impact of B vitamins on global cognition remains inconclusive.

Data from the NHANES 2011–2014 cohort indicated that higher intake of vitamin B1 was significantly associated with better global cognitive performance in adults aged 60 years and older [49]. However, a 2021 meta-analysis by Zhang et al. found no consistent cognitive benefit from high B-vitamin intake in cross-sectional studies [65].

Similarly, a large RCT by Kang et al., involving more than 2000 older women with cardiovascular risk factors, showed no significant cognitive benefit from long-term supplementation with B6, B12, and folic acid over a 5.4-year period [69].

Some meta-analyses suggest that B-vitamin supplementation—particularly when administered for longer than three months—may help slow cognitive decline in non-demented populations [66,67,95], but not in individuals with dementia [68]. Nonetheless, other meta-analyses have reported no significant cognitive effects [17,70,71], although folate may confer greater benefits than B6 or B12 [71].

In conclusion, while some data support a potential cognitive benefit of B-vitamin supplementation in non-demented older adults, particularly with longer treatment duration, the overall evidence remains inconsistent, especially among populations with existing cardiovascular or cognitive conditions.

#### 3.3.2. Omega-3 LC-PUFAs and Global Cognition

Evidence regarding the effects of omega-3 LC-PUFAs on global cognition is mixed.

Some RCTs such as that by Stavrinou et al. [52], reported significant improvements in global cognition in older adults with MCI following high-dose omega-3 and antioxidant supplementation. Similarly, in healthy older adults, Ichinose et al. [72] observed improved MMSE scores with DHA supplementation. However, larger trial in older adults [76] did not find significant cognitive effects.

In a specific trial among overweight and obese adults, omega-3 supplementation did not outperform a weight-loss diet alone in improving global cognition [77].

Meta-analyses also provide conflicting results. While Zhang et al. [73], Brainard et al. [74], and Yang et al. [75] identified small but significant benefits on global cognition, other reviews, including those by He et al. [78], Alex et al. [19] and Kosti et al. [53], found no meaningful effect. Fairbairn et al. [15] reported positive effects of multinutrient supplementation—including omega-3 and B vitamins—on global cognition, especially when assessed using composite neuropsychological scores.

A 2023 scoping review by Andriambelo et al. [96], encompassing 78 RCTs, found that 44% of studies reported cognitive benefits with omega-3 supplementation, particularly in MCI populations (67% showing improvement). However, substantial variability in dosages, populations, and outcome measures limits firm conclusions.

In summary, while some evidence supports the cognitive benefits of omega-3 LC-PUFAs—particularly in MCI populations—findings across trials remain inconsistent. Further standardized, large-scale RCTs are needed to determine their role in enhancing or preserving global cognition.

#### 3.3.3. Antioxidants and Global Cognition

Observational evidence strongly supports a protective role of antioxidant-rich diets on global cognitive function.

In a prospective study of 960 older adults, higher flavonol intake was associated with significantly slower cognitive decline over nearly a decade, independent of other lifestyle factors [24]. Similarly, in the NHANES dataset, older adults with higher oxidative balance scores—reflecting high antioxidant intake and low pro-oxidant exposure—demonstrated better global cognitive performance [56]. A cross-sectional study by Godos et al. in Southern Italy further found that greater total flavonoid intake correlated with better cognitive status among older adults [79].

Findings from RCTs further reinforce these observations. In the COSMOS trial, daily multivitamin–multimineral (MVM) supplementation over three years led to improved global cognitive scores, particularly in individuals with cardiovascular comorbidities and lower baseline cognitive performance [31]. When pooling data from multiple COSMOS cognitive sub-studies, the MVM group showed cognitive benefits equivalent to a delay in cognitive aging by approximately two years [31]. However, no significant effects were seen from cocoa extract supplementation over a two-year period [34].

Other trials also support the cognitive benefits of specific antioxidants. A 24-month intervention in postmenopausal women using resveratrol improved a composite cognition score [30]. Supplementation with carotenoids [80] and dietary flavonoids [81] was also associated with significant improvements in global cognitive performance.

However, not all antioxidants show benefit; for example, selenium supplementation failed to improve cognitive function in a 2022 meta-analysis by Pereira et al. [82].

Collectively, evidence from observational studies and several randomized trials indicates that diets rich in antioxidants—particularly multivitamins, flavonoids, carotenoids, and resveratrol—may support global cognitive health in older adults, although efficacy appears to vary by compound and population. An important nuance is that cognitive domains may not improve uniformly: some multicomponent trials find global and memory benefits but no change in executive function, which suggests that the **global composite gains are often driven by memory**. Nonetheless, preserving global cognition is the ultimate goal, and antioxidants—especially in broad combinations—show promise in **preventing global cognitive decline** in at-risk older adults.

#### 3.3.4. MIND Diet and Global Cognition

Observational studies consistently suggest that higher adherence to the MIND diet is associated with better global cognitive function across diverse populations. For instance, participants in the China Health and Nutrition Survey who adhered more closely to the MIND diet performed better on global cognition measures—approximately equivalent to being one year younger in age [83]. Similarly, a prospective U.S. study in older adults found that those in the highest tertile of adherence showed significantly slower decline in global cognition over eight years [84]. Other cohorts, such as the Puerto Rican Health Study [85] and the Framingham Offspring cohort [61], have reported positive associations between MIND adherence and baseline MMSE or cognitive composite scores. A small Iranian RCT combining the MIND diet with physical activity also reported meaningful gains in MMSE over a three-month intervention [87]. However, not all findings are positive: data from the UK Biobank (~120,000 adults) showed no benefit—and even slightly worse cognitive scores—in those with higher MIND adherence, suggesting that benefits may not be detectable in younger or well-nourished populations [88]. Nonetheless, the bulk of longitudinal studies indicate a positive link between MIND diet adherence and cognitive preservation [86], suggesting that the MIND diet may contribute to cognitive resilience in the elderly.

RCTs, on the other hand, have provided less convincing results. The largest trial to date found no significant difference in global cognition between MIND diet and control groups (both of which received mild caloric restriction) [89]. Similarly, a 12-week trial in middle-aged adults found no significant improvements in global cognitive performance [41].

Recent meta-analyses indicate that a one-standard deviation increase in MIND diet score is associated with better global cognition [83], although some meta-analyses have not confirmed significant effects [90].

The MIND diet shows consistent observational links with better global cognitive function, but randomized trials have yet to confirm a definitive benefit, emphasizing the need for further high-quality, long-duration studies.

#### 3.3.5. Ketogenic and Low-Carbohydrate Diets and Global Cognition

Emerging data suggest that ketogenic interventions may benefit global cognition, particularly in older adults and individuals with AD. A meta-analysis of 10 RCTs [91] demonstrated significant improvements in MMSE and ADAS-Cog scores following 3 to 15 months of ketogenic dietary interventions in AD patients. Complementary findings from an RCT in frail elderly adults showed a 3.5-point MMSE increase after three months of MCT supplementation, reinforcing the cognitive potential of ketone-based approaches [92].

Ketogenic diets and MCT supplementation show promising effects on global cognition in aging and AD populations, although larger and longer-term studies are needed to confirm these benefits across broader groups.

#### 3.3.6. Dietary Restrictions (CR and IF) and Global Cognition

Emerging evidence suggests that dietary restriction strategies may offer modest benefits for global cognitive function, particularly in older adults with MCI.

A large longitudinal study found that older adults with MCI who regularly practiced IF exhibited better global cognition scores [46]. A meta-analysis further supported these findings, reporting the strongest cognitive improvements in individuals with MCI, followed by normal-weight individuals and those who were overweight [48]. Additionally, a recent pilot study demonstrated that an eight-week prolonged nightly fasting regimen (14 h per night) led to significant improvements in global cognition among older adults with subjective cognitive complaints, suggesting potential benefits of circadian-aligned eating patterns on cognitive health [93].

In populations with obesity or metabolic risk, results are more variable. A four-week trial in sedentary older adults using a daily 16-h IF regimen found no significant change in MoCA scores [94]. Similarly, Keawtep et al. [47] reported no global cognitive improvements after three months of IF in postmenopausal women with obesity. However, Hugenschmidt et al. [64] observed a modest improvement in MMSE scores after adding moderate or high CR to a 20-week aerobic exercise program.

While findings are mixed, dietary restriction strategies—particularly when implemented in individuals with MCI—may confer modest benefits to global cognitive performance, though results in metabolically compromised populations remain inconsistent. Further long-term RCTs are warranted to determine the optimal conditions for cognitive benefit.

### 3.4. Mental Health

Depression is a frequent mood disorder that combines emotional, physical, and cognitive symptoms. The cognitive domains most impacted by depression are attention, memory and learning, executive function, and psychomotor processing. It is also a key neuropsychiatric comorbidity in cognitive aging: up to one-third of individuals with depression present with MCI, and depression significantly increases the risk of progression to dementia, particularly AD.

While traditionally viewed through a neurochemical lens, depression is now understood as a multifactorial condition involving psychological, genetic, and inflammatory mechanisms. Chronic low-grade inflammation is increasingly recognized as a shared pathway linking depression with metabolic and neurodegenerative diseases. Diet, by modulating inflammation and neurobiological processes, may therefore influence both the onset and trajectory of depressive symptoms [97] (Table 4).

#### 3.4.1. B Vitamins and Mental Health

Observational data indicate that regular intake of B vitamin-fortified foods may reduce depression risk [109].

However, evidence from randomized controlled trials remains inconsistent. While one trial in older adults with MCI showed short-term improvements in depressive symptoms with B12 and folate supplementation, this benefit was not sustained over time [51] and a meta-analysis of RCTs found no overall effect of vitamin B12 supplementation on depressive symptoms [17].

Multinutrient combinations including B vitamins have shown modest improvements in anxiety and mental fatigue in healthy populations, but the overall effect of B vitamins on mood remains limited and context-dependent [16].

#### 3.4.2. Omega-3 LC-PUFAs and Mental Health

Omega-3 fatty acids have shown potential for improving depressive symptoms, particularly in specific subgroups.

While early trials suggested benefits in middle-aged women [98] and individuals with anxiety [99], larger RCTs like the VITAL-DEP study [101] failed to demonstrate a significant effect of omega-3 supplementation on preventing major depressive disorder in high-risk older adults. A recent meta-analysis indicates that the efficacy of omega-3s may depend on the type and dose, with DHA and low-dose EPA showing benefits, particularly in patients with MCI [100].

Overall, evidence remains mixed, and further well-powered trials are needed to clarify their role in mental health.

#### 3.4.3. Antioxidants and Mental Health

Antioxidant-rich diets and supplements have shown promise in reducing depressive symptoms, particularly in older adults.

Evidence from RCTs suggests that curcumin supplementation can significantly improve depression and anxiety scores when used alongside standard treatments for major depressive disorder [102].

Although findings from smaller studies, such as the blueberry intervention in memory-impaired older adults, are less conclusive. They observed a trend toward reduced depressive symptoms with daily blueberries but this was not statistically robust (*p* = 0.08) due to the small sample [26].

Overall, antioxidant interventions appear to have potential as adjunctive strategies for mood improvement, particularly in populations vulnerable to oxidative stress.

#### 3.4.4. MIND Diet and Mental Health

Beyond cognitive performance, the MIND diet has been investigated for its impact on mental health outcomes such as depression.

Observational studies indicate that higher adherence to the MIND diet is generally associated with fewer depressive symptoms and reduced psychological distress in older adults and diverse populations [103,104]. However, one study found no association between adherence to the MIND diet and incident depression, while a significant association was observed with adherence to the Mediterranean diet [105]. Longitudinal data suggest this relationship persists over time, even after adjusting for lifestyle factors, although not all cohorts—particularly healthier, younger populations—show consistent effects. A few RCTs also suggest that short-term adherence to the MIND diet may improve mood outcomes [41].

While causality remains to be confirmed, current evidence supports a potential role for the MIND diet in promoting mental well-being, particularly in populations at risk of depression.

#### 3.4.5. Ketogenic Diet and Mental Health

Preliminary studies suggest that KDs may offer mental health benefits, particularly in individuals with mood disorders.

Observational and retrospective data indicate that KD adherence is associated with improved emotional well-being, reduced stress and anxiety, and decreased symptoms of depression and psychosis [106], including in treatment-refractory populations [107]. Evidence from patients with bipolar disorder also suggests potential for greater mood stabilization compared to other dietary interventions [108].

Despite these findings, high-quality RCTs are lacking, and further research is needed to determine the clinical efficacy, long-term effects, and relapse risk post-diet discontinuation [110]. Ongoing trials are currently investigating the impact of KD on treatment-resistant depression, bipolar disorder, and schizophrenia [111,112].

## 4. Discussion

This comprehensive review examined the effects of various dietary interventions—ranging from specific nutrients to broader dietary patterns—on cognitive outcomes and mental health in older adults. The findings indicate that diet can exert measurable influence on memory, executive function, global cognition, and mood, particularly in populations at risk of cognitive decline.

Memory appears to be the most consistently improved domain across nutritional interventions. Multinutrient approaches combining B vitamins and omega-3 PUFAs show synergistic effects on episodic memory, especially in individuals with low baseline performance or metabolic risk factors. Antioxidant-rich foods and polyphenol supplementation also demonstrate promising benefits for memory preservation. The MIND diet, in particular, is associated with enhanced memory performance in cognitively healthy adults, although evidence remains scarce in MCI or dementia populations.

Executive function, however, shows more heterogeneous responses. While higher intake of B vitamins (particularly B1 and B2), omega-3 PUFAs, and antioxidants is associated with better executive functioning in observational studies, randomized trials yield mixed results. The variability in dosage, intervention duration, and population characteristics may explain these discrepancies. Diets such as the MIND or ketogenic diet show limited but emerging support for executive function improvement, primarily in individuals with MCI or obesity when combined with other interventions (physical activity or nitrate-rich foods).

When assessing global cognition, multivitamin and antioxidant supplementation (especially in trials such as COSMOS) demonstrate small but statistically significant effects. Similarly, meta-analyses suggest that ketogenic and omega-3-based interventions may offer cognitive protection in AD or MCI populations, although findings remain inconsistent. The MIND diet shows the strongest observational support for global cognition, yet RCTs fail to confirm definitive benefits, likely due to methodological limitations such as short duration or control diet overlap.

Finally, mental health outcomes—particularly depression—are increasingly recognized as both a modifiable risk factor for cognitive decline and important endpoints in their own right. Omega-3 fatty acids (particularly DHA and low-dose EPA), curcumin, and antioxidant-rich diets may improve mood or reduce depressive symptoms, although findings are more robust in individuals with existing psychological distress. The MIND diet also appears to support mood stability, and ketogenic diets have shown promise in treatment-resistant depression, although data from high-quality RCTs are still needed.

Beyond cognitive performance, dietary factors may influence the onset and progression of neurodegenerative disorders, particularly dementia [113]. Epidemiological data consistently suggest that higher adherence to healthy dietary patterns, especially the Mediterranean and MIND diets, is associated with a lower risk of dementia. Specific nutrients such as omega-3 polyunsaturated fatty acids, B vitamins, polyphenols, and carotenoids have been proposed as key mediators of these effects, based on both observational findings and mechanistic studies. This reinforces the concept that nutrition is a modifiable risk factor for cognitive decline and dementia, particularly when implemented from midlife or earlier. The 2020 Lancet Commission estimated that up to 40% of dementia cases could be prevented through the modification of lifestyle factors, including dietary behaviors [9].

Although causality has yet to be demonstrated through well-designed, long-term randomized controlled trials, current evidence strongly supports the integration of nutritional strategies within multidomain preventive approaches for dementia.

Despite these encouraging signals, the overall quality of evidence remains variable, with most trials suffering from limited sample sizes, short follow-up periods, and heterogeneous cognitive outcomes. Consequently, current guidelines (WHO and ESPEN [7,114]) do not recommend routine supplementation or specific dietary protocols for cognitive preservation in dementia, citing insufficient evidence of efficacy. These recommendations highlight the importance of caution and the need for further research before widespread implementation of dietary interventions in clinical settings.

## 5. Conclusions

As an overview, this review intends to offer clinicians and researchers a synthesized framework of current knowledge, highlighting promising dietary strategies and identifying areas where further investigation is critically needed.

In summary, dietary interventions, especially multinutrient supplementation and antioxidant-rich patterns such as the MIND diet, may contribute to preserving memory and global cognition in older adults, particularly those with early cognitive decline or metabolic comorbidities. Although evidence remains variable for executive function and mental health, certain subgroups appear to benefit.

However, given the complexity of cognitive aging, a holistic approach is essential. Nutrition alone is unlikely to be sufficient; integrated strategies that combine physical activity, vascular risk control, cognitive stimulation, and social engagement are more likely to promote lasting cognitive resilience.

Future research should prioritize these holistic, multimodal strategies, examining how nutritional interventions can be effectively combined with other lifestyle and medical approaches to reduce dementia risk across diverse populations. In light of the growing interest in the gut–brain axis, future studies should also explore how dietary interventions interact with the microbiome to influence brain health and cognitive aging.

## Figures and Tables

**Table 1:** Recent data examining the effects/associations of various dietary patterns, vitamins and nutrients on memory.

	Observational		Interventional	
	Positive	Negative	Positive	Negative
**Vitamins**	**Zhou [12]**: 2023, older adults (NHANES database), higher vitamin B2 intake = better verbal memory (statistically significant) **Gillies [13]**: 2023, secondary analysis of the REACH Study, older adults, higher glycine concentration = better episodic memory (*p* = 0.016)		**Yeung [14]**: 2023, older adults, (COSMOS-Web trial), multivitamin supplementation vs. placebo = improve episodic memory at 1 year (*p* = 0.025), and at 3 years (*p* = 0.011) **Fairbairn [15]**: 2023, meta-analysis, B vitamins + *n*-3 LC-PUFA = improve episodic memory (*p* = 0.001)	**Young [16]**: 2022, middle-aged adults, multinutrient formula containing B vitamins 12 weeks = no benefit to memory (*p* = 0.470) **Markun [17]**: 2021, meta-analysis, no significant effect of B12 on memory
**Omega-3 long-chain** **polyunsaturated fatty acids**	**Wang [18]:** 2024, older adults (NHANES), omega-3 PUFA intake = better verbal memory (β = 0.53, *p* < 0.0001)		**Alex [19]:** 2020, omega-3 LC-PUFA = benefits on memory function (*p* = 0.003) **Maltais [20]:** 2022, healthy adults, EPA + DHA 6 months = improved episodic memory in low baselines scorers (*p* = 0.043) **Malik [21]**: 2021, older adults with coronary artery disease, EPA + DHA 30 months = improve memory (RAVLT) at 12 & 30 months (*p* = 0.047) **Fairbairn [15]**: 2023 meta-analysis, B vitamins + *n*-3 LC-PUFA = improve episodic memory (*p* = 0.001).	**Van de Rest [22]**: 2008, older adults, EPA + DHA (fish oil) 26 weeks = no effect on memory (*p* > 0.05) **Dangour [23]**: 2010, older adults, EPA + DHA 24 months = no effect on memory (*p* > 0.05)
**Antioxidants**	**Holland [24]:** 2023, community-dwelling, flavonol intake = slower decline in episodic memory (β = 0.004 (95% CI 0.002–0.006)) **Kesse-guyot [25]**: 2012: French adults, antioxidant mix = better verbal memory 13 years later (*p* = 0.01)		**Krikorian [26]**: 2010, older adults with memory complaints, blueberry juice = improved paired-associate learning (*p* = 0.009) and recall (*p* = 0.04) **Lopresti [27]:** 2024, middle-aged adults with subjective memory complaints, vitamin E + astaxanthin + grape juice extract = improve episodic memory (*p* = 0.037) **Bensalem [28]**: 2019, older adults, grape and blueberry polyphenols 6 months = improve verbal episodic memory in individuals with lower baseline performance **Nouchi [29]:** 2023, older adults, 6-MSITC (wasabi extract) 12 weeks = significant improvement in episodic memory **Thaung Zaw [30]:** 2021, postmenopausal women with metabolic syndrome, resveratrol = relative improvement in verbal memory vs. younger than 65 years **Vyas [31]:** 2024, meta-analysis (cosmos), multivitamin-mineral supplementation = benefits on episodic memory (*p* = 0.0007) **Zhu [32]**, 2019, meta-analysis (6 RCTs; healthy older adults, AD, schizophrenia patients), Curcumin = improved memory in healthy older adults (*p* = 0.02) **De Vries [33]:** 2022, meta-analysis polyphenols = improve episodic memory (*p* < 0.001)	**Vyas [34]**: 2024, (cosmos), cocoa extract 2 years = no significant effects in episodic memory [mean difference (95% CI): −0.01 (−0.13, 0.10) SU] **Curtis [35]**: 2024, MCI, American elderberry juice 12 weeks = no significant improvement in memory (*p* > 0.05) **Khorshidi [36]:** 2021, meta-analysis, RSV = no significant effect on memory performance
**MIND diet**	**Wesselman [37]:** 2021, older adults, greater adherence to MIND diet = better memory (*p* = 0.029) **Calil [38]:** 2018, older adults control group, greater adherence to MIND diet = better verbal memory (*p* < 0.05) **Berendsen [39]**: 2018, women aged 70+, greater adherence to MIND diet = better verbal memory (*p*-trend = 0.006)	**Calil [38]:** 2018, MCI/AD, no diet–cognition correlation	**Arjmand [40]:** 2022, healthy obese women, MIND diet group = improve verbal recognition memory vs control group (*p* < 0.05)	**Timlin [41]**: 2025, RCT—Feasibility, midlife adults, MIND diet 12 weeks = no measurable change in memory performance
**Ketogenic diet**	**Wang [42]**: 2022, older adults (NHANES), LCD score = better verbal memory		**Krikorian [43]**: 2012, MCI, LCD 6 weeks = improve verbal memory (*p* = 0.01) **Fortier [44]**: 2019, MCI, kMCT 6 months = episodic memory improved versus baseline	**Buchholz [45]**: 2024, AD patients, 12-weeks modified Atkins diet vs. control diet = non-significant improvement in memory
**Caloric restriction/** **Intermittent fasting**	**Ooi [46]:** 2020, MCI, practicing IF (regularly or not) = increment in episodic memory (RAVLT) upon 36 months of follow-up (*p* < 0.001)		**Keawtep [47]**: 2024, obese women, obesity, IF + exercise = improvement in episodic memory (*p* < 0.05) but not in the diet group alone (*p* > 0.05) **Lu [48]**: 2023, meta-analysis, dietary restriction = improve memory SMD = 0.18 (95% CI: 0.00–0.35, *p* = 0.05)	

AD: Alzheimer’s disease; CI: confidence interval; DHA: docosahexaenoic acid; EPA: eicosapentaenoic acid; IF: intermittent fasting; kMCT: ketogenic medium chain triglyceride; LCD: Low-carbohydrate-diet; MCI: mild cognitive impairment; MIND: Mediterranean-Dietary Approaches to Stop Hypertension (DASH) Intervention for Neurodegenerative Delay; 6-MSITC: 6-(Methylsulfinyl)hexyl isothiocyanate; NHANES: National Health and Nutrition Examination Survey; OR: odds ratio; PUFA: Polyunsaturated fatty acids; RAVLT: Rey-Auditory Verbal Learning Test; RCTs: randomized clinical trials; RSV: Resveratrol; SMD: standardized mean difference.

**Table 2:** Recent data examining the effects/associations of various dietary patterns, vitamins and nutrients on executive functions.

	Observational		Interventional	
	Positive	Negative	Positive	Negative
**Vitamins**	**Jia [49]:** 2024, older adults (NHANES), Higher vitamin B1 intake = higher DSST/AFT scores (*p* < 0.001) **Zhou [12]:** 2023, older adults (NHANES), the highest quartile of vitamin B2 intake = 45.1-fold increase on the DSST test sores (*p* = 0.004)		**Zhou [50]:** 2023, MCI, B12 6 months = improvements in calculation (*p* < 0.01)	**Kwok [51]:** 2020, MCI, methylcobalamin 500 μg + folic acid 400 μg once daily 12 months = improve executive function (*p* = 0.004), but effects at month 24 not significant **Markun [17]:** 2021, meta-analysis, no effect of B12 alone and B complex supplementation on executive function (*p* = 0.82)
**Omega-3 long-chain** **polyunsaturated fatty acids**	**Wang [18]:** 2024, older adults (NHANES), dietary omega-3 PUFA intake = better DSST (*p* = 0.0045)		**Stavrinou [52]:** 2020, MCI, Omega-3/6 PUFA + antioxidant vitamins 6 months = improve executive function (Stroop Color Test, *p* = 0.037) **Kosti [53]:** 2022, meta-analysis, changes in EPA and DHA body status = positive impact on participants’ executive functions	**Howe [54]:** 2018, mildly hypertensive older adults, fish oil (DHA + EPA) 20 weeks = no significant effect on executive functions **Andrieu [55]:** 2017, older adults with memory complaints, DHA 3 years = no significant effect on DSST (*p* > 0.05)
**Antioxidants**	**Song [56]:** 2023, older adults (NHANES), oxidative balance score = better DSST (the beta estimates (95% CI) 0.009 (0.002, 0.025)) **Mao [57]:** 2024, older adults (NHANES), increased CDAI = better executive function (*p* < 0.05), more pronounced in stroke patients			**Curtis [35]:** 2024, MCI, American elderberry juice 12 weeks = trends of improved in cognitive flexibility but not significant (*p* = 0.09) **Vyas [34]**: 2024 (cosmos), cocoa extract = no significant effect on executive function [mean difference (95% CI): 0.003 (−0.07, 0.08) SU] **Nouchi [29]:** 2023, older adults, 6-MSITC (wasabi extract) 12 weeks = no significant improvement in executive functions **Kesse-guyot [25]:** 2012, French adults, antioxidant mix (polyphenol) = no improvement with executive functioning (*p* = 0.09) **Zhu [32]:** 2019, meta-analysis (6 RCTs), healthy older adults, AD and schizophrenia patients, curcumin = no significant improvement in executive function **Ammar [58]:** 2020, meta-analysis, polyphenol rich diet = no significant effect on executive functions **Morton [59]:** 2021, meta-analysis, fruit derived polyphenol = no significant effect on executive function **Farag [60]:** 2022, meta-analysis, polyphenols = no significant effect on executive function
**MIND diet**	**Melo van Lent [61]:** 2021, community-based population, higher adherence to MIND diet = better performance in executive function (*p* = 0.01)	**Nishi [62]:** 2021, overweight/obese and metabolic syndrome middle aged adults, baseline MIND diet scores = no significant association with change in executive function		**Arjmand [40]:** 2022, healthy obese women, MIND diet 3 months = no statistically significant effect on TMT-B (*p* = 0.161)
**Ketogenic diet**		**Wang [42]:** 2022, older adults (NHANES), LCD score = no association with executive function (AFT, DSST)	**Fortier [44]:** 2019, MCI, kMCT 6 months vs. placebo = modest improvement of inhibitory capacity post-treatment	
**Caloric restriction/** **Intermittent fasting**			**Keawtep [47]:** 2024, obese women, CR + exercise= improvement in TMT B = (*p* < 0.05) But not in the diet group alone (*p* > 0.05) **Alharbi [63]:** 2023, overweight /obese middle-aged and older adults, CR +/− beetroot juice 14 days = improve TMT-B (*p* = 0.002) with greater improvements observed in the CR + beetroot juice vs.CR alone (*p* = 0.012)	**Hugenschmidt [64]:** 2019, obese older adults, adding CR + to a 20-week aerobic exercise program 24 months = no improvement in executive function more than exercise alone

AD: Alzheimer’s disease; AFT: animal fluency test; CDAI: composite Dietary Antioxidant Index; CI: confidence interval; CR: caloric restriction; DHA: docosahexaenoic acid; EPA: eicosapentaenoic acid; kMCT: ketogenic medium chain triglyceride; LCD: Low-carbohydrate-diet; MCI: mild cognitive impairment; MIND: Mediterranean-Dietary Approaches to Stop Hypertension (DASH) Intervention for Neurodegenerative Delay; 6-MSITC: 6-(Methylsulfinyl)hexyl isothiocyanate; NHANES: National Health and Nutrition Examination Survey; OR: odds ratio; PUFA: Polyunsaturated fatty acids; RCTs: randomized clinical trials; RSV: Resveratrol; TMT-B: Trail making test B.

**Table 3:** Recent data examining the effects/associations of various dietary patterns, vitamins and nutrients on global cognition.

	Observational		Interventional	
	Positive	Negative	Positive	Negative
**Vitamins**	**Jia [49]:** 2024, older adults (NHANES), higher vitamin B1 intake = higher global cognition z sore (β = 0.09, 95% CI 0.02~0.16)	**Zhang [65]**: 2021, meta-analysis, no benefit in global cognition for high B vitamins intake	**Suh [66]:** 2020, meta-analysis, middle-aged or older adults, B vitamins 3 months = improve global cognition (SMD = −0.18, 95% CI = −0.30 to −0.06) **Li [67]:** 2021, meta-analysis, MCI or cognitively healthy elderly adult, vitamin B supplements = improve global cognitive (*p* < 0.01) **Wang [68]:** 2022, meta-analysis, B vitamins = improve cognitive function (MMSE) (mean differences = 0.14, 95%CI = 0.04–0.23) **Fairbairn [15]:** 2023, metanalysis, B vitamins + *n*-3 PUFA = improve global cognition (*p* = 0.002)	**Kang [69]:** 2008, women ≥ 40 y + high risk CV, B vitamin vs. placebo groups = no difference in global score (*p* = 0.30) **Behrens [70]**: 2020, meta-analysis, B vitamins = no significant effect on cognitive function (*p* = 0.39) **Markun [17]:** 2021, meta-analysis, B12 alone = no significant effect on cognitive function **Chang [71]:** 2023, meta-analysis, vitamin B supplements = no significant effect on MMSE
**Omega-3 long-chain** **polyunsaturated fatty acids**			**Stavrinou [52]:** 2020, MCI,| Omega-3/6 PUFA + antioxidant vitamins 6 months = improve global cognition (MMSE, *p* = 0.011) **Ichinose [72]**: 2021, older adults, EPA + DHA 12 months = Higher MMSE score in DHA group vs. placebo (*p* < 0.05) **Zhang [73]:** 2020, omega-3 LC-PUFA = improve global cognition (WMD = 0.85, 95% CI = 0.04–1.67) **Brainard [74]**: 2020, omega-3 LC-PUFA = improve MMSE (MD 0.10, 95% CI 0.03–0.16) **Yang [75]:** 2023, DHA or/and EPA = benefits on global cognition (SMD = 0.51, 95% CI = 0.12–0.91) **Fairbairn [15]**: 2023, metanalysis, B vitamins + *n*-3 PUFA = improve global cognition (*p* = 0.002)	**Danthiir [76]:** 2018, older adults, DHA + EPA 18 months = no significant effect on global cognition (*p* > 0.05) **Salman [77]:** 2022, overweight/obese adults, EPA + DHA + other omega 3PUFAs = no additional benefit on global cognition (MoCA) **Alex [19]:** 2020, omega-3 LC-PUFA = no effect on global cognitive function (Hedge’s g = 0.02, 95% CI = −0.12–0.154) **He [78]:** 2023, meta-analysis (15 RCTs), older adults, *n*-3 PUFA = no overall effect on MMSE (*p* = 0.53) **Kosti [53]:** 2022, meta-analysis, changes in EPA and DHA body status = no impact on overall cognitive performance
**Antioxidants**	**Holland [24]:** 2023, community-dwelling, flavonol intake = slower decline in global cognition (β estimate = 0.004 (95% CI 0.001–0.006)) **Song [56]:** 2023, older adults (NHANES), oxidative balance score = positively associated with global cognitive function (β estimates (95% CI) 0.030 (0.024, 0.074)) **Godos [79]:** 2020, adults living in southern Italy, higher total flavonoid intake = better cognitive status (OR ≈ 0.39 for highest vs. lowest quartile, 95% CI 0.15–1.00))		**Vyas [31]:** 2024, meta-analysis (cosmos), multivitamin-mineral supplementation = benefits on global cognition (*p* = 0.0009) **Thaung Zaw [30]:** 2021, postmenopausal women, trans-resveratrol 12 months (crossover design) = 33% improvement in overall cognitive performance (*p* = 0.005) **Davinelli [80]**: 2021, carotenoids = improve cognitive functions (*p* < 0.0001) **Cheng [81]**: 2022, dietary flavonoids = improve cognitive performance (*p* < 0.001)	**Vyas [34]:** 2024, (cosmos), cocoa extract 2 years = no significant effect in global cognition **Pereira [82]:** 2022, meta-analysis, selenium = no significant effect on cognitive function (MMSE & ADAS-Cog tests)
**MIND diet**	**Huang [83]:** 2023, middle-aged and older adults, MIND diet scores = positively associated with global cognitive function (*p*-trend < 0.001) + meta-analysis: 1 SD increment of MIND score = +0.042 units (95% CI = 0.020–0.065) in global cognitive function z-score **Agarwal [84]:** 2024, older adults (biracial), higher MIND diet score = slower cognitive decline (*p* trend = 0.0025) **Boumenna [85]:** 2022, older Puerto Rican adults, the highest versus lowest MIND quintile = better cognition function (*p* trend = 0.0019) **Melo van Lent [61]:** 2021, community-based population, higher MIND diet scores = better global cognitive function (*p* = 0.004) **Dhana [86]:** 2021, older adults, higher MIND diet score = better global cognitive functioning proximate to death (*p* = 0.003) **Ahn [87]:** 2022, older adults (NHANES), adherence to MIND diet + exercise = better global cognition (*p* < 0.001)	**Cornelis [88]**: 2022, middle-aged adults, top vs. bottom MIND quintile: worse baseline scores on global cognition (mean differences on z-score scale ~–0.02 to –0.05, *p* < 0.002)		**Barnes [89]:** 2023, older adults + family history of AD, MIND diet 3 years = no differences in global cognition vs. control group (*p* = 0.23) **Timlin [41]:** 2025, RCT-Feasibility, healthy midlife adults, MIND diet 12 weeks = no significant differences in cognitive function between groups **Fu [90]**: 2022, MIND diet = no significant improvement on global cognition
**Ketogenic diet**			**Rong [91]:** 2024, meta-analysis, AD patients (10 RCTs), KD 3 to 15 months = improve MMSE scores (*p* = 0.002) and ADAS-Cog scores (*p* = 0.008) **Abe [92]:** 2020, frail elderly, kMCT supplementation 3 months = 3.5-point increase in MMSE scores (*p* < 0.001)	
**Caloric restriction/** **Intermittent fasting**	**Ooi [46]:** 2020, MCI practicing IF (regularly or not) = better MMSE score (*p* < 0.001)		**Lu [48]:** 2023, dietary restriction = improve global cognitive function (*p* < 0.05) **James [93]:** 2024, pilot study, older adults, 8-week regimen of prolonged nightly fasting (14 h per night) = improve cognitive function (*p* = 0.02)	**Keawtep [47]:** 2024, obese women, no improvement in MoCA score for each group (CR, CR + exercise, control)(*p* 0.687) **Anton [94]:** 2019, older overweight adults, IF 4 weeks = no significant change in cognitive

AD: Alzheimer’s disease; ADAS-Cog: Assessment Scale-Cognitive subscale; CI: confidence interval; CR: caloric restriction; DHA: docosahexaenoic acid; EPA: eicosapentaenoic acid; IF: intermittent fasting; kMCT: ketogenic medium chain triglyceride; MCI: mild cognitive impairment; MIND: Mediterranean-Dietary Approaches to Stop Hypertension (DASH) Intervention for Neurodegenerative Delay; MMSE: Mini-Mental State Examination; MoCA: Montreal Cognitive Assessment; NHANES: National Health and Nutrition Examination Survey; PUFA: Polyunsaturated fatty acids; RCTs: randomized clinical trials; SD: standard deviation; SMD: standardized mean difference.

**Table 4:** Recent data examining the effects/associations of various dietary patterns, vitamins and nutrients on mental health.

	Observational		Interventional	
	Positive	Negative	Positive	Negative
**Vitamins**	**Moore [94]:** 2019, community-dwelling adults, B vitamin-fortified foods if consumed daily = reduced risk of depression (OR 0.54, 95% CI 0.41–0.70)		**Young [16]:** 2022, middle aged adults, multinutrient containing B group vitamins 12 weeks = lower state anxiety and mental fatigue in those with an ‘optimal’ diet	**Kwok [51]:** 2020, MCI, methylcobalamin 500 μg + folic acid 400 μg once daily 24 months = lower HDRS score at 12 months (*p* = 0.012) but effects at month 24 not significant **Markun [17]:** 2021, meta-analysis, B12 alone and B complex supplementation = no overall effect of vitamin supplementation on measures of depression
**Omega-3 long-chain** **polyunsaturated fatty acids**			**Freeman [98]:** 2011, middle-aged women/menopausal transition, EPA + DHA 8 weeks = decrease in MADRS scores (*p* < 0.0001) **Aucoin [99]:** 2024, adult women with generalized anxiety disorder, dietary counseling + omega-3 12 weeks = decrease in anxiety symptoms **Chang [100]:** 2024, meta-analysis, patients with MCI and dementia, DHA = significantly reduced depressive symptoms (*p* = 0.039) + low doses of EPA = significantly effective (*p* = 0.001), with MCI (*p* < 0.001).	**Chang [100]:** 2024, meta-analysis, patients with MCI and dementia, EPA + DHA = non-significant trend toward affecting depressive symptoms (*p* = 0.141) **Vyas [101]:** 2023, older adults, vitamin D3 + omega-3 fatty acids 2 years = no significant differences in PHQ-9 score vs. placebo.
**Antioxidants**			**Panahi [102]**: 2015, patients with major depressive disorder, standard antidepressive therapy + curcuminoids–piperine combination = greater reductions in total HADS score vs. control group (*p* < 0.001)	**Krikorian [26]**: 2010, older adults with memory complaints, blueberry juice = trends suggesting reduced depressive symptoms but not significant (*p* = 0.08)
**MIND diet**	**Cherian [103]:** 2021, older adults, highest tertile of the MIND diet scores = lower rates of depressive symptoms (β = −0.12, CI: −0.23, −0.0092) **Kamrani [104]:** 2024, participants in the highest quartile of MIND diet = lower risks of depression (*p* = 0.005) and anxiety (*p* = 0.008)	**Fresan [105]**: 2019, adults free of depression, no association of the MIND diet and incident depression	**Timlin [41]**: 2025, RCT—Feasibility, midlife adults, MIND diet 12 weeks = improved mood (*p* < 0.05)	
**Ketogenic diet**	**Garner [106]:** 2024, general healthy population, KD = higher self-reported mental and emotional well-being behaviors **Danan [107]:** 2022, adults with severe, persistent mental illness, KD = associated with improvements in depression and psychosis symptoms (*p* < 0.001) **Campbell [108]:** 2019, individuals with bipolar disorder, KD = association with mood stabilization or remission of symptoms over a period (*p* < 0.0001)			

CI: confidence interval; DHA: docosahexaenoic acid; EPA: eicosapentaenoic acid; HADS: Hospital Anxiety and Depression Scale; HDRS: Hamilton depression rating scale; MADRS: Montgomery–Asberg Depression Rating Scale; MCI: mild cognitive impairment; MIND: Mediterranean-Dietary Approaches to Stop Hypertension (DASH) Intervention for Neurodegenerative Delay; OR: odds ratio; PHQ-9: Patient Health Questionnaire-9; PUFA: Polyunsaturated fatty acids; RCT: randomized clinical trial.

## List of Abbreviations

6-MSITC: 6-(Methylsulfinyl)hexyl isothiocyanate, AD: Alzheimer’s disease, ADAS-Cog: Alzheimer’s Disease Assessment Scale–Cognitive Subscale, AFT: Animal Fluency Test, ALA: alpha-linolenic acid, CERAD memory test: Consortium to Establish a Registry for Alzheimer’s Disease memory test, BRJ: rich beetroot juice, CR: caloric restriction, COSMOS-Web trial: the COcoa Supplement and Multivitamin Outcomes Study Web, DASH diets: Dietary Approaches to Stop Hypertension, DHA: docosahexaenoic acid, DNA: deoxyribonucleic acid, DSST: Digit Symbol Substitution Test, EPA: eicosapentaenoic acid, ESPEN: European Society for Clinical Nutrition and Metabolism, IF: intermittent fasting, KD: Ketogenic diet, MAD: the modified Atkins diet, the MAPT study: The Multidomain Alzheimer Preventive Trial, MCI: mild cognitive impairment, MCT: medium-chain triglyceride, MIND diet: Mediterranean-DASH diet intervention for neurological delay, MMSE: Mini-Mental State Examination, MoCA: Montreal Cognitive Assessment, ModRey test: the Modified Rey Auditory Verbal Learning Test, MVM: multivitamin-multimineral, NHANES: National Health and Nutrition Examination Survey, PREDIMED-Plus cohort: PREvención con DIeta MEDiterránea-Plus trial, PUFAs: Omega-3 long-chain polyunsaturated fatty acids, RAVLT: the Rey Auditory Verbal Learning Test, RCTs: Randomized controlled trials, REACH cohort: the Researching Eating, Activity, and Cognitive Health cohort, U.S.: United States, VITAL-DEP study: VITamin D and OmegA-3 TriaL-Depression Endpoint Prevention study, WHO: World Health Organization.
